# Impact of pulmonary hypertension on long-term neurodevelopmental outcomes in preterm neonates with bronchopulmonary dysplasia: A meta-analysis

**DOI:** 10.12669/pjms.42.7.16628

**Published:** 2026-07

**Authors:** Yonghong Peng, Juanya Yang

**Affiliations:** 1 Yonghong Peng, Department of Neonatology, Huzhou Maternity & Child Health Care Hospital, Huzhou, Zhejiang Province 313000, P.R. China; 2 Juanya Yang, Department of Neonatology, Huzhou Maternity & Child Health Care Hospital, Huzhou, Zhejiang Province 313000, P.R. China

**Keywords:** Bronchopulmonary dysplasia, Pulmonary hypertension, Neurodevelopment, Risk

## Abstract

**Objective:**

To systematically evaluate the association between pulmonary hypertension (PH) and long-term neurodevelopmental outcomes in preterm infants with bronchopulmonary dysplasia (BPD).

**Methodology:**

A systematic review and meta-analysis were conducted in accordance with PRISMA guidelines. PubMed, Embase, Web of Science, and Scopus were searched from inception to January 28, 2026 for observational studies comparing preterm infants with BPD with and without PH and reporting neurodevelopmental outcomes assessed beyond 12 months of corrected age. Random-effects meta-analyses were performed for continuous outcomes (cognitive, language, and motor scores; standardized mean difference [SMD]) and dichotomous adverse neurodevelopmental outcomes (odds ratio [OR]).

**Results:**

Seven studies were included. Four studies contributed continuous outcome data. PH was associated with significantly lower cognitive (SMD −0.47, 95% CI −0.81 to −0.14), language (SMD −0.28, 95% CI −0.50 to −0.05), and motor scores (SMD −0.50, 95% CI −0.73 to −0.28). Heterogeneity for continuous outcomes was low (I² = 0%). Five studies provided extractable dichotomous data, demonstrating that PH was associated with a higher risk of adverse neurodevelopmental outcomes (OR 2.80, 95% CI 1.57–4.98), with moderate heterogeneity (I² = 48.8%).

**Conclusions:**

PH in preterm infants with BPD may be associated with poorer long-term neurodevelopmental outcomes. Further studies are needed to improve the evidence.

***Registration No:*** PROSPERO (CRD420261296106).

## INTRODUCTION

Bronchopulmonary dysplasia (BPD) is a common long-term complication seen with prematurity.^1^ Evidence shows that BPD affects not only impacts the lungs but also cardiovascular health, physical growth, and neurodevelopment.^1^,^2^ Infants with moderate to severe BPD are prone to long-lasting adverse effects due to prolonged respiratory support, systemic inflammation, and disruption of organ development. Pulmonary hypertension (PH) is a key complication of BPD. It occurs mostly due to abnormal pulmonary blood vessel growth and maladaptive vascular changes in the immature lung tissue.^3^ PH suggests a high-risk BPD subtype and is associated with worse respiratory function, longer oxygen dependence, and higher mortality.^4^

In addition to the effects on respiratory and cardiovascular systems, BPD-associated PH may also affect neurodevelopment of patients. It is suggested that chronic hypoxemia, impaired pulmonary perfusion, and prolonged exposure to the illness during the time of development may cause adverse neurodevelopmental outcomes.^3^,^5^ Some cohort studies^6^,^7^ involving preterm infants with BPD have shown that children with concurrent PH may have poorer neurodevelopmental performance in early childhood, affecting cognitive, motor, as well as language domains. This is particularly important for nursing personnel as they are closely associated with long-term monitoring of such patients. Nurses can detect early warning signs and help select children at risk for closer follow-up.

While research has shown some evidence on the association between BPD-related PH and adverse neurodevelopment, individual studies have shown variability in sample size, outcome definitions, and neurodevelopmental assessment methods. A few studies have used continuous developmental scores, while others have categorize neurodevelopmental impairment dichotomously. This has complicated direct comparisons across studies.^8^-^10^ Therefore, this systematic review and meta-analysis was conducted to assess the available evidence on the relationship between PH and long-term neurodevelopmental outcomes in preterm neonates with BPD.

## METHODOLOGY

This systematic review and meta-analysis was conducted in accordance with the Preferred Reporting Items for Systematic Reviews and Meta-Analyses (PRISMA) guidelines.^11^ The study protocol was prospectively registered on PROSPERO (CRD420261296106).

Two reviewers independently conducted a thorough literature search across PubMed, Embase, Web of Science, and Scopus. Search was from the inception of each database up to January 28, 2026. We imposed no language or date restrictions. The complete electronic search strategy is shown in Supplementary Table-I. Additionally, we also checked the reference lists of included studies and pertinent reviews to identify additional eligible studies. Studies were selected based on the Population, Exposure, Comparator, and Outcome (PECO) framework.

**Supplementary Table-I:** Search strategy.

***PubMed (MEDLINE)***
(“Bronchopulmonary Dysplasia”[Mesh] OR bronchopulmonary dysplasia OR BPD) AND (“Pulmonary Hypertension”[Mesh] OR pulmonary hypertension OR PH) AND (“Infant, Premature”[Mesh] OR preterm OR premature OR very preterm OR extremely preterm) AND (“Neurodevelopmental Disorders”[Mesh] OR neurodevelopment* OR neurodevelopmental outcome* OR neurodevelopmental impairment OR developmental delay OR cognitive outcome* OR motor outcome* OR language outcome* OR Bayley OR “Bayley Scales”).
***Embase***
(‘bronchopulmonary dysplasia’/exp OR bronchopulmonary dysplasia OR BPD) AND (‘pulmonary hypertension’/exp OR pulmonary hypertension OR PH) AND (‘premature infant’/exp OR preterm OR premature OR extremely preterm) AND (‘neurodevelopmental disorder’/exp OR neurodevelopment* OR neurodevelopmental outcome* OR neurodevelopmental impairment OR developmental delay OR cognitive outcome* OR motor outcome* OR language outcome* OR bayley).
***Web of Science***
TS=((“bronchopulmonary dysplasia” OR BPD) AND (“pulmonary hypertension” OR PH) AND (preterm OR premature OR “very preterm” OR “extremely preterm”) AND (neurodevelopment* OR neurodevelopmental outcome* OR neurodevelopmental impairment OR developmental delay OR cognitive outcome* OR motor outcome* OR language outcome* OR bayley)).
***Scopus***
TITLE-ABS-KEY ((“bronchopulmonary dysplasia” OR BPD) AND (“pulmonary hypertension” OR PH) AND (preterm OR premature OR “very preterm” OR “extremely preterm”) AND (neurodevelopment* OR neurodevelopmental outcome* OR neurodevelopmental impairment OR developmental delay OR cognitive outcome* OR motor outcome* OR language outcome* OR bayley).

### Population

Preterm infants diagnosed with BPD.

### Exposure

Presence of PH diagnosed by standard criteria.

### Comparator

Infants without PH.

### Outcomes

Long-term neurodevelopmental outcomes assessed at ≥12 months corrected age, including cognitive, language, or motor development.

We included both observational cohort studies and secondary analyses of randomised trials. Studies were excluded if they did not provide separate data for PH versus non–PH groups, reported outcomes only during hospitalization, or lacked sufficient data for quantitative synthesis. Case reports and animal studies were also excluded.

### Inclusion & Exclusion Criteria:

All retrieved records were imported into the reference management software. Duplicate records were removed electronically. Two independent reviewers then screened the titles and abstracts of all records to assess their relevance to the clinical question. Articles which seemed potentially eligible after the initial screening underwent a thorough full-text review. Any disagreements between reviewers were resolved via discussion.

### Data extraction:

Data extraction was performed independently by two reviewers. The variables extracted were: author, year of publication, country of study, study design, sample size, gestational age, severity, PH definition, age at neurodevelopmental assessment, assessment instrument used, and reported neurodevelopmental outcomes. For continuous variables, the mean and standard deviation (SD) of cognitive, language, and motor scores were extracted from the studies. When studies presented data as medians with interquartile ranges or ranges, they were converted into mean and SD.^12^ For dichotomous outcomes, the number of infants with adverse neurodevelopmental outcomes and the total number sample size was extracted.

### Risk of bias assessment:

The quality of the studies was judged using the Newcastle–Ottawa Scale.^13^ It has three domains: selection of participants, comparability between groups, and outcomes assessment. Studies with scores >7 were marked as moderate to high quality. Assessment was carried out independently by two reviewers, and any disagreements were resolved through discussion.

### Statistical analysis:

Meta-analyses were conducted using random-effects models. For continuous neurodevelopmental outcomes, we generated standard mean differences (SMDs) with 95% confidence intervals (CIs) i.e. for cognitive, language, and motor scores. Negative SMD values suggested worse neurodevelopmental performance in infants with PH. For dichotomous outcomes, we calculated odds ratios (ORs) with 95% CIs to compare the risk of adverse neurodevelopmental outcomes between the PH and non–PH groups. Studies without separate PH–stratified data were excluded from this analysis. Heterogeneity was assessed using Cochran’s Q test and the I² statistic, with I² values of 0–25% considered low, 26–50% moderate, and over 50% substantial heterogeneity. A two-sided p value <0.05 was deemed statistically significant. All analyses were performed using ‘R’ software.

## RESULTS

**Fig.1:**
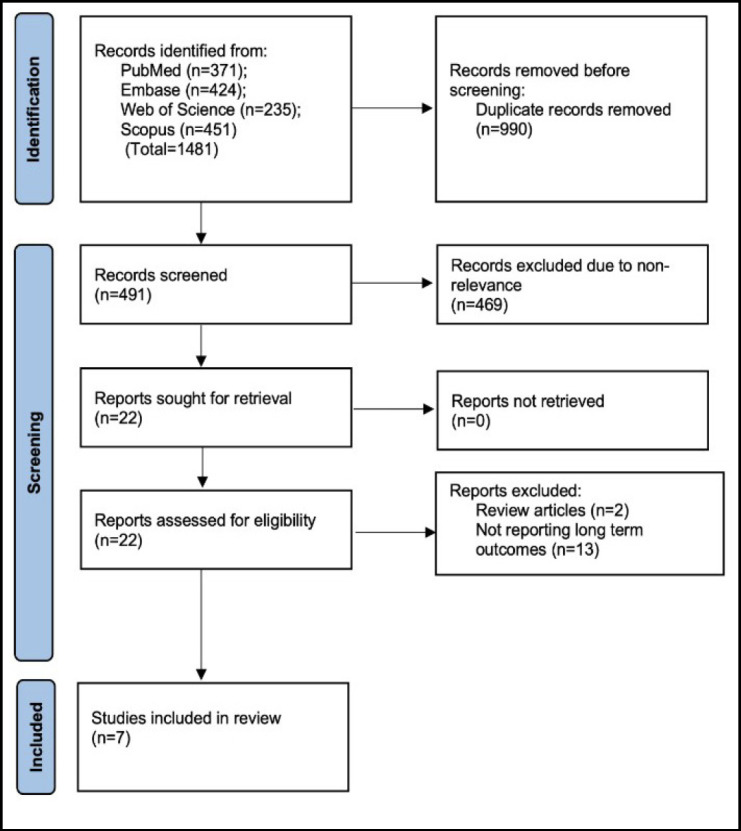
Study flowchart.

The study flowchart is shown in Fig.1. The search of all databases led to 1481 articles. After excluding duplicates, 491 studies remained. Twenty-two studies were selected for full-text analysis. Seven fulfilled the inclusion criteria.^6^-^10^,^14^,^15^ There were no disagreements between the reviewers for inclusion of the studies.

The included studies were published between 2016 to 2024. There were six retrospective cohort studies and one prospective cohort study (Table-I). The studies were from Japan, South Korea, Canada, the United Kingdom, and the United States. All studies were on very or extremely preterm infants, with gestational ages < 28 weeks or <32 weeks. PH was mostly diagnosed via echocardiography. One study diagnosed late-onset PH as one needing pharmacological intervention beyond 36 weeks’ of corrected age. Neurodevelopmental follow-up assessments were conducted at or beyond 18 months of corrected age in all studies. In one cohort study, the follow-up was three years. Sample sizes varied considerably. Neurodevelopmental outcomes were evaluated using validated instruments, mostly using the Bayley Scales of Infant and Toddler Development (Bayley-III). Other tools used were the Kyoto Scale of Psychological Development and composite measures that integrated standardized screening instruments. The risk of bias analysis of the included studies is shown in Supplementary Table-II. Three studies received a score of six, three received a score of seven, and only one received a score of nine.

**Table-I:** Details of included studies.

Study Country	Des-ign	Population	PH definition	Follow-up	Sample size PH	Sample size no-PH	Gestational age PH (wks)	Gestational age no-PH (wks)	Tool used & definition of adverse outcome
Nakanishi, 2016 Japan	RC	<28 wk preterm with BPD	Echo PH	3 years	20	60	24.8±1.3	25.7±1.4	KSPD; DQ<70
Choi, 2019 South Korea	RC	<28 wk preterm with moderate–severe BPD	Echo PH	18–24 m	20	61	25.3±1.4	25.8±1.1	Bayley-III; <85
MacKenzie, 2020 Canada	RC	<32 wk preterm with moderate–severe BPD	Echo PH	18–22 m	24	63	NR	NR	Bayley-III; <85
Baczynski, 2021 Canada	RC	<32 wk preterm with moderate–severe BPD	Echo PH	18–24 m	46	74	NR	NR	Bayley-III
Thomas, 2023 Canada/USA	RC	<29 wk preterm with moderate–severe BPD	Echo PH	18–24 m	51	199	NR	NR	Bayley/ASQ; <85
Branescu, 2024 UK	RC	≤32 wk preterm with moderate–severe BPD	Echo PH	24 m	19	182	NR	NR	Bayley-III
Kim, 2024 South Korea	PC	22–27 wk with severe BPD	Late PH requiring medications >36wk	18–24 m	38	185	25.0±1.4	25.1±1.4	Mixed

RC, retrospective cohort; PC, prospective cohort; BPD, Bronchopulmonary dysplasia; Echo, echocardiography; m, months; PH, pulmonary hypertension; wk, weeks; KSPD, Kyoto Scale of Psychological Development; NR, not reported; ASQ, Ages and Stages Questionnaire version 3; DQ, developmental quotient.

**Supplementary Table-II:** Risk of bias analysis

*Study*	*Selection*	*Comparability*	*Outcome*	*Total*
Nakanishi 2016	★★★☆ (3)	★☆ (1)	★★☆ (2)	6/9
Choi 2019	★★★☆ (3)	★☆ (1)	★★☆ (2)	6/9
MacKenzie 2020	★★★☆ (3)	★★ (2)	★★☆ (2)	7/9
Baczynski 2021	★★★☆ (3)	★★ (2)	★★☆ (2)	7/9
Thomas 2023	★★★☆ (3)	★★ (2)	★★☆ (2)	7/9
Branescu 2024	★★★☆ (3)	★☆ (1)	★★☆ (2)	6/9
Kim 2024	★★★★ (4)	★★ (2)	★★★ (3)	9/9

**Fig.2:**
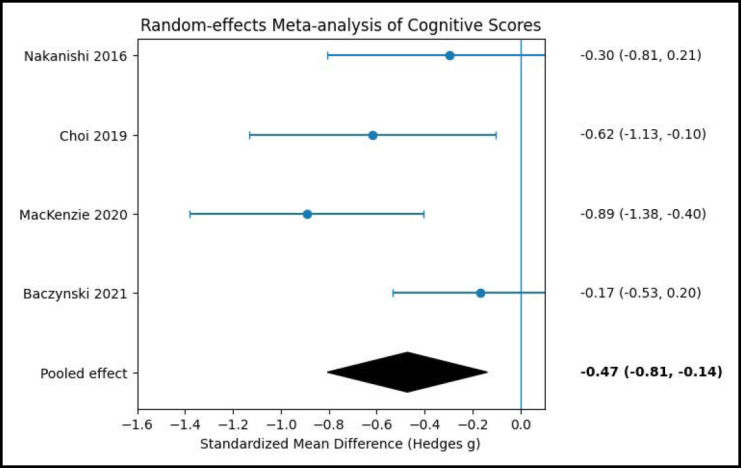
Meta-analysis of cognitive scores between PH and non-PH groups with BPD.

**Fig.3:**
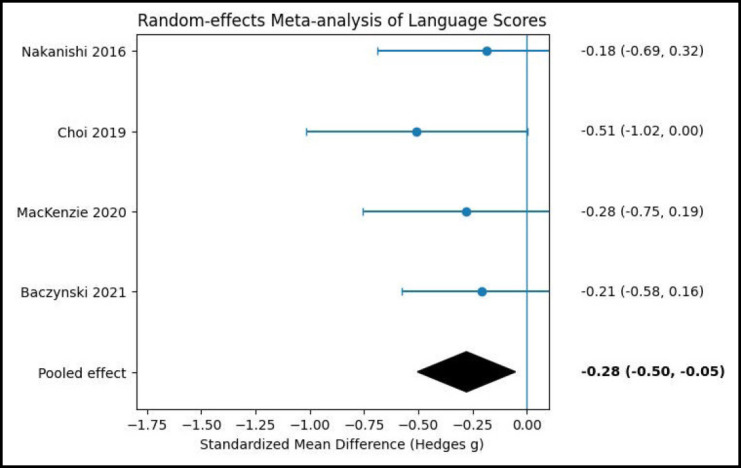
Meta-analysis of language scores between PH and non-PH groups with BPD.

**Fig.4:**
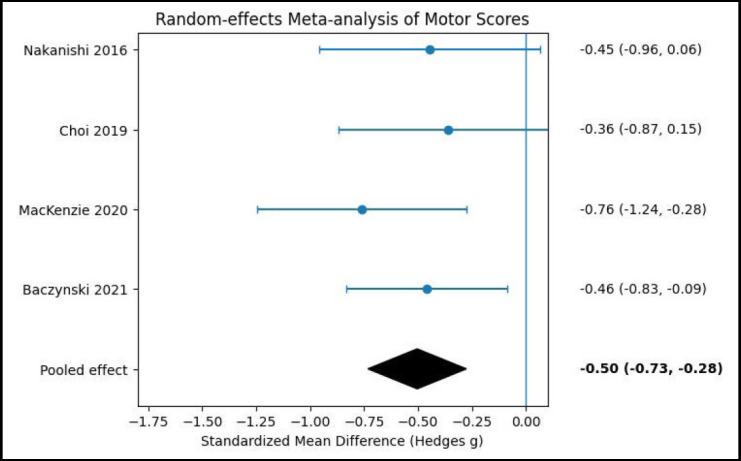
Meta-analysis of motor scores between PH and non-PH groups with BPD.

Four studies reported data for meta-analysis of cognitive outcomes. Infants with PH had significantly lower cognitive scores compared to those without PH (SMD −0.47, 95% CI −0.81 to −0.14, I^2^=0%) (Fig.2). All included studies showed similar results, with lower average cognitive scores in the PH group. Four studies reported data on the language outcomes analysis. The meta-analysis showed a significant reduction in language scores among infants with PH versus those without PH (SMD −0.28, 95% CI −0.50 to −0.05, I^2^=0%) (Fig.3). The direction of this outcome was also consistent across studies. Data on motor outcomes were reported by four studies. The pooled analysis showed that PH was associated with significantly lower motor scores (SMD −0.50, 95% CI −0.73 to −0.28, I^2^=0%) (Fig.4). All studies showed poorer motor performance in the PH group.

**Fig.5:**
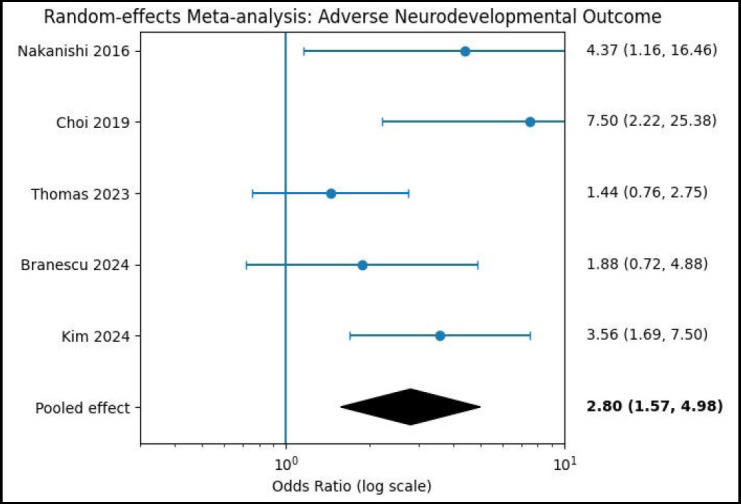
Meta-analysis of risk of adverse neurodevelopmental outcome between PH and non-PH groups with BPD.

Five studies provided PH–stratified dichotomous data on adverse long-term neurodevelopmental outcomes. Meta-analysis showed that the presence of PH was associated with a significantly increased risk of adverse neurodevelopmental outcomes compared to no PH (OR 2.80, 95% CI 1.57 to 4.98, I^2^=49%) (Fig.5).

## DISCUSSION

This systematic review and meta-analysis showed a significant association between PH and poor long-term neurodevelopmental outcomes in preterm infants with BPD. Meta-analysis of continuous data showed that infants with PH had significantly lower cognitive, language, and motor scores compared to those without PH. Meta-analysis of dichotomous data also supported these results and showed that PH was linked with a higher risk of adverse neurodevelopmental outcomes. Low heterogeneity was noted in continuous data meta-analysis indicating some reliability of findings.

PH is a serious complication associated with BPD. It signifies the close relationship between impaired lung development and pulmonary vascular disease in extremely preterm infants.^16^ A previous meta-analysis has shown that PH occurs in about 20% of preterm infants with BPD. The prevalence also increases significantly as the severity of the condition worsens, reaching nearly 40% in severe BPD.^17^ In BPD, disrupted formation of alveoli is accompanied by abnormal growth of pulmonary blood vessels, decreased vascular density, and adverse vascular remodelling. These changes lead to increased resistance in the pulmonary vessels and higher pulmonary arterial pressures. It can be worsened by prolonged mechanical ventilation, oxygen toxicity, and inflammatory injury. Such abnormalities in the pulmonary blood vessels lead to respiratory and heart-related events, increased risk of death, low oxygen levels, and changes in overall blood flow.^2^,^17^ A number of risk factors related to the perinatal period, birth, and growth have been identified for the development of PH in infants with BPD. These include oligohydramnios, lower gestational age, lower birth weight, small for gestational age status, neonatal respiratory distress syndrome, sepsis, and severe BPD. On the other hand, exposure to antenatal steroids may reduce this risk.^18^

PH with BPD has been shown to affect short-term clinical outcomes in infants. In a recent review,^2^ infants with PH and BPD showed significantly higher rates of in-hospital complications, like mortality, prolonged respiratory support, higher dependence on supplemental oxygen, and need for pulmonary vasodilator therapy. These short-term adverse outcomes suggest that pulmonary vascular disease has a major effect on lung gas exchange, right heart function, and overall health progression in infants with advanced BPD.^2^ While early cardiovascular and respiratory effects of PH in BPD have been noted, its long-term effects beyond the neonatal period are not completely understood. Building on these short-term findings, the current study further adds that PH in preterm infants with BPD may also be linked with worse long-term neurodevelopmental outcomes, including lower scores in cognitive, language, and motor domains.

In comparison, other literature also supports an association between PH and adverse long-term neurodevelopmental outcomes. A prospective, population-based cohort study using the EPIPAGE-2 data has shown that extremely and very preterm children with prior history of neonatal PH exhibited significantly higher incidences of moderate to severe neurodevelopmental disabilities at five years of age. These included higher rates of cerebral palsy, cognitive impairments, and developmental coordination disorders vs preterm peers without PH (36.9% versus 17.9%, P<0.001).^19^ Another study from term and near-term children with persistent PH of the newborn, has shown high prevalence of developmental abnormalities. Nearly 50% of children had scored within pathological ranges of at least one domain of the Ages and Stages Questionnaire at follow-up period of 1-5 years and there were impairments noted in communication and personal–social skills.^20^ A retrospective, single-center study^21^ conducted on infants born before 30 weeks’ gestation has noted that higher grades of BPD (Grades 2 and 3), which more frequently co-occurred with late PH, were significantly associated with higher risk of adverse neurodevelopmental outcomes. These findings show that PH may lead to severity-dependent neurodevelopmental risks, particularly in children with advanced BPD.

There are various mechanisms by which PH may contribute to the development of adverse outcomes in BPD. Current evidence shows that PH associated with BPD affects the pulmonary vascular development, leading to impaired angiogenesis, decreased capillary density, endothelial dysfunction, and abnormal smooth muscle cell proliferation.^3^ These vascular changes can cause an increase in pulmonary vascular resistance and lead to persistent pressure on the right ventricle, thereby compromising cardiopulmonary reserve and leading to chronic hypoxemia.^4^,^22^ Prolonged hypoxemia, with fluctuations in cerebral oxygen supply during key stages of brain development, could adversely affect neuronal growth, myelination, and synapse formation.^5^,^23^ Furthermore, extended invasive ventilation, oxygen toxicity, and systemic inflammation which is often required in infants with severe BPD and PH, can worsen neuroinflammatory responses and oxidative stress, leading to brain injury.^3^,^5^,^24^ Several studies also suggest that changes in systemic perfusion and reduced cardiac output in these infants might limit cerebral blood flow, thereby increasing the risk of ischemic or hypoxic damage.^19^,^20^

### Limitations

Most studies were observational in nature which limits our ability to establish causality. The present results should therefore be interpreted as only an association between the two. Moreover, we cannot exclude the risk of residual confounding. Infants with PH are a subgroup with more severe underlying BPD and therefore with greater overall clinical instability. Factors like prolonged mechanical ventilation, extended oxygen exposure, recurrent hypoxemia, systemic inflammation, sepsis, impaired growth, and other prematurity-related comorbidities may independently contribute to adverse neurodevelopmental outcomes. Therefore, PH may in part function as a marker of overall disease severity of BPD rather than an isolated risk factor of impaired neurodevelopment. The assessment of PH neurodevelopmental outcomes also differed among studies. There were differences in the instruments and definitional criteria employed and could lead to heterogeneity and misclassification bias. Additionally, several continuous variables were converted from median to means and SD, potentially introducing calculation errors. Overall, only seven studies were available in the literature and data on dichotomous PH-stratified events was available only from a subset of studies, thereby reducing the statistical power. Another limitation is that only crude data was pooled and adjusted estimates were not available across studies. Lastly, data on critical variables like timing and severity of PH, treatment protocols, and long-term follow-up beyond early childhood, were scarce. This prevented a more detailed subgroup analyses.

### Strength of the study:

It is the most updated review assessing long-term neurodevelopmental impact of PH in BPD patients. A thorough literature search was conducted to include seven recent publications. The findings of this meta-analysis indicate that PH can be a marker to identify a high-risk subgroup of preterm infants with BPD who may have worse neurodevelopmental outcomes in the long term. Both physicians and nursing personnel can be involved in early detection and systematic surveillance for PH in infants with moderate to severe BPD. Such patients may benefit from more thorough cardiopulmonary management and require regular screening for future neurodevelopmental impairments. Multidisciplinary follow-up strategies involving physicians, pulmonologists, nursing personnel that combine cardiopulmonary monitoring with early developmental assessments and interventions, may improve long-term functional outcomes in such patients.

## CONCLUSIONS

PH in preterm infants with BPD seems to be associated with significantly poorer long-term neurodevelopmental outcomes, including lower cognitive, language, and motor scores, as well as a higher risk of neurodevelopmental impairment. However, due to the observational nature of the evidence and the potential of residual confounding, these findings should not be interpreted as establishing a direct causal effect of PH on neurodevelopment. Further research is needed to strengthen the findings.

### Authors’ contributions:

**YP:** Literature search, study design and manuscript writing.

**YP and JY:** Data collection, data analysis and interpretation. Critical Review.

**YP:** Manuscript revision and validation and is responsible for the integrity of the study.

All authors have read and approved the final manuscript.

## References

[1] Maia PD, Abman SH, Mandell E (2024). Bronchopulmonary Dysplasia-Associated Pulmonary Hypertension: Basing Care on Physiology. Neoreviews.

[2] Mascarenhas D, Al-Balushi M, Al-Sabahi A, Weisz DE, Jain A, Jasani B (2025). Pulmonary hypertension in preterm neonates with bronchopulmonary dysplasia: a meta-analysis. Arch Dis Child Fetal Neonatal.

[3] El-Saie A, Varghese NP, Webb MK, Villafranco N, Gandhi B, Guaman MC (2023). Bronchopulmonary dysplasia - associated pulmonary hypertension: An updated review. Semin Perinatol.

[4] Mani S, Mirza H, Ziegler J, Chandrasekharan P (2024). Early Pulmonary Hypertension in Preterm Infants. Clin Perinatol.

[5] Volpe JJ (2009). Brain injury in premature infants: a complex amalgam of destructive and developmental disturbances. Lancet Neurol.

[6] Nakanishi H, Uchiyama A, Kusuda S (2016). Impact of pulmonary hypertension on neurodevelopmental outcome in preterm infants with bronchopulmonary dysplasia: a cohort study. J Perinatol.

[7] Choi EK, Shin SH, Kim EK, Kim HS (2019). Developmental outcomes of preterm infants with bronchopulmonary dysplasia-associated pulmonary hypertension at 18-24 months of corrected age. BMC Pediatr.

[8] Baczynski M, Kelly E, McNamara PJ, Shah PS, Jain A (2021). Short and long-term outcomes of chronic pulmonary hypertension in preterm infants managed using a standardized algorithm. Pediatr Pulmonol.

[9] Thomas SR, Jain SK, Murthy P, Joseph CJ, Soraisham A, Tang S (2024). Neurodevelopmental Outcomes of Preterm Infants Born <29 Weeks with Bronchopulmonary Dysplasia-Associated Pulmonary Hypertension: A Multicenter Study. Am J Perinatol.

[10] Kim C, Kim S, Kim H, Hwang J, Kim SH, Yang M (2024). Long-term impact of late pulmonary hypertension requiring medication in extremely preterm infants with severe bronchopulmonary dysplasia. Sci Rep.

[11] Page MJ, McKenzie JE, Bossuyt PM, Boutron I, Hoffmann TC, Mulrow CD (2021). The PRISMA 2020 statement: an updated guideline for reporting systematic reviews. BMJ.

[12] Wan X, Wang W, Liu J, Tong T (2014). Estimating the sample mean and standard deviation from the sample size, median, range and/or interquartile range. BMC Med Res Methodol.

[13] Lo CK, Mertz D, Loeb M (2014). Newcastle-Ottawa Scale: comparing reviewers' to authors' assessments. BMC Med Res Methodol.

[14] MacKenzie K, Cunningham K, Thomas S, Mondal T, El Helou S, Shah PS (2020). Incidence, risk factors, and outcomes of pulmonary hypertension in preterm infants with bronchopulmonary dysplasia. Paediatr Child Health.

[15] Branescu I, Alexandru DO, Vladareanu S, Kulkarni A (2024). Two-Year Outcomes in Preterm Infants Suffering from Moderate to Severe Bronchopulmonary Dysplasia with or without Associated Pulmonary Hypertension. Curr Health Sci J.

[16] Ramanand P, Indic P, Gentle SJ, Ambalavanan N (2025). Detection of pulmonary hypertension in preterm infants with bronchopulmonary dysplasia using oxygen saturation data. Pediatr Res.

[17] Arjaans S, Zwart EAH, Ploegstra MJ, Bos AF, Kooi EMW, Hillege HL (2018). Identification of gaps in the current knowledge on pulmonary hypertension in extremely preterm infants: A systematic review and meta-analysis. Paediatr Perinat Epidemiol.

[18] Li B, Qu SS, Li LX, Zhou N, Liu N, Wei B (2024). Risk factors and clinical outcomes of pulmonary hypertension associated with bronchopulmonary dysplasia in extremely premature infants: A systematic review and meta-analysis. Pediatr Pulmonol.

[19] Breinig S, Ehlinger V, Rozé JC, Storme L, Durrmeyer X, Cambonie G (2025). Neurodevelopmental outcomes at age 5 years among children born very preterm and surviving after persistent pulmonary hypertension of the newborn: EPIPAGE-2 cohort study. Europ J Paediatr Neurol.

[20] Atlan L, Berthomieu L, Karsenty C, Gascoin G, Arnaud C, Breinig S (2024). Neurodevelopmental outcome in children between one and five years after persistent pulmonary hypertension of term and near-term newborns. Front Pediatr.

[21] Oluwole I, Tan JBC, DeSouza S, Hutchinson M, Leigh RM, Cha M (2023). The association between bronchopulmonary dysplasia grade and risks of adverse neurodevelopmental outcomes among preterm infants born at less than 30 weeks of gestation. J Matern Fetal Neonatal Med.

[22] Rasheed J, Khalid M, Nawaz I, Maryam B (2024). Echocardiographic evaluation of myocardial dysfunction in term neonates with perinatal asphyxia. Pak J Med Sci.

[23] Tran NN, Votava-Smith JK, Wood JC, Panigrahy A, Wee CP, Borzage M (2021). Cerebral oxygen saturation and cerebrovascular instability in newborn infants with congenital heart disease compared to healthy controls. PLoS One.

[24] Kalteren WS, Verhagen EA, Mintzer JP, Bos AF, Kooi EMW (2021). Anemia and Red Blood Cell Transfusions, Cerebral Oxygenation, Brain Injury and Development, and Neurodevelopmental Outcome in Preterm Infants: A Systematic Review. Front Pediatr.

